# Procalcitonin Use for Predicting Mortality and Morbidity of Patients Diagnosed With Sepsis Within the Intensive Care Unit

**DOI:** 10.7759/cureus.48080

**Published:** 2023-10-31

**Authors:** Christopher R Chew, Dhaval Patel, Ania I Rynarzewska, Louise Jones

**Affiliations:** 1 Critical Care, Louisiana State University (LSU) Shreveport, Shreveport, USA; 2 Critical Care, Northeast Georgia Medical Center Gainsville, Gainesville, USA; 3 Marketing, Georgia College and State University, Milledgeville, USA; 4 Research, Northeast Georgia Medical Center Gainsville, Gainesville, USA

**Keywords:** morbidity, mortality, procalcitonin, severe sepsis, septic shock management, medical intensive care unit (micu)

## Abstract

Objective

Infections leading to severe sepsis and septic shock are among the top five causes requiring admission to the intensive care unit (ICU). Up to 40% of ICU admissions contain a sepsis diagnosis. Without a clear marker to diagnose and manage sepsis, procalcitonin has been extensively studied for its usefulness in the management of bacterial infections. These studies, however, have been focused toward how it can be used to help guide when antibiotics should be initiated and de-escalated. There, however, has not been a study on how this biomarker could be used to predict mortality, and morbidity and help guide a need for antibiotic escalation.

Design

A retrospective chart review was conducted on patients admitted to the ICU at Northeast Georgia Medical Center between January 1, 2019, to June 30, 2021. Inclusion criteria were all patients above the age of 18 admitted to the ICU with a diagnosis of sepsis and having at least two procalcitonin drawn within 10 days of each other. Exclusion criteria were any patient with a diagnosis of COVID-19.

Data

Analysis was conducted to identify how delta procalcitonin could identify mortality and morbidity and if there was any change in antibiotics based on the delta procalcitonin.

Conclusion

There was a statistically significant association between a delta positive procalcitonin and increased ICU length of stay. There was no statistical significance in expiration based on the antibiotic change in relationship to delta positive change in procalcitonin.

## Introduction

Infections leading to severe sepsis and septic shock are among the top five causes requiring admission to the intensive care unit (ICU). Up to 40% of ICU admissions contain a sepsis diagnosis [1]. Not all infections are obvious or bacterial in nature. Various lab markers, such as ESR, CRP, and procalcitonin, have been developed to assist clinicians in diagnosing and managing infections. Despite this, not one marker has been shown to be the gold standard in predicting infection or outcome.

Procalcitonin (pro-cal) is a lab test that has been developed to identify the presence and severity of bacterial infections [1]. It has been initially well-studied in its use toward bacterial infections of the lung [2]. More recently, it has been studied as a means of de-escalating antibiotics in the setting of sepsis given the high sensitivity toward bacterial infections has allowed for antibiotic stewardship to prevent the rising crisis of multi-drug resistant organisms [3]. The current accepted pro-cal levels of 0.25 and 0.5 ug/L are concerning for a likely bacterial infection, and greater than 0.5 ug/L is very suggestive of a bacterial infection to help guide the necessity for antibiotics [1]. Shehabi et al. demonstrated pro-cal’s usefulness in guiding antibiotic decision-making in ICU patients in their literature review with a significant negative predictive value (99%) and a specificity of 99% for systemic infections [1].

A follow-up randomized clinical trial performed by Shehabi et al. [4] further examined pro-cal and detection of infection/sepsis in over 400 patients within 11 ICUs in Australia. The target population was those with suspected bacterial infections and the expectation of receiving antibiotics with an ICU stay for at least 24 hours [4]. While there was not a significant reduction in antibiotic use, ventilation time, hospital stay, or ICU stay between the pro-cal-guided and standard treatment arms, it is noted by the study that 0.1 ug/L is used as the cutoff level to determine antibiotic use or no antibiotic use [4]. Of note, there was a demonstration of a faster decline in procalcitonin over the first 72 hours with appropriate antibiotic use, which demonstrated a relationship with higher survivability [4]. Other notable findings included elevated pro-cal levels greater than 3 ug/L were highly sensitive for a positive culture with pro-cal levels being most significantly elevated in blood cultures, followed by urine and respiratory cultures [4].

A breakthrough trial of the use of procalcitonin was a non-inferiority randomized controlled trial performed by Bouadma et al. [5].  The trial was a 630-patient trial, with 311 assigned to the pro-cal group and 319 to the standard treatment group [5]. The patients assigned to the pro-cal group had antibiotics used sparingly and were stopped based on the cutoff level of 0.5 ug/L [5]. Of note, however, the antibiotics used and the decision to ultimately stop was left at the discretion of the physician [5]. The significance of the trial demonstrated that the mortality of the pro-cal guided group was non-inferior to those in the control group at day 28 and day 60, and the exposure to antibiotics was significantly less in the pro-cal guided group compared to the standard treatment group, with a difference of 2.7 [5].

As noted by the article included in the Cleveland Clinic Journal by Fakheri [6], PRO-RATA was a randomized controlled trial of 630 ICU patients, and antibiotic use was encouraged in patients with a pro-cal level greater than 0.5 ng/mL [6]. Another pro-cal randomized control trial, the SAPS trial (2013), with 1,816 participants, demonstrated a decrease in antibiotic use and a lower mortality rate using pro-cal as guidance for antibiotic use [7].

## Materials and methods

The design of this study was a retrospective chart review. This study adopts the definition of "critically ill patient” from Robertson and Al-Haddad: Critical illness is a life-threatening multisystem process that can result in significant morbidity or mortality. The selection criteria were retrospective studies that focused on patients who were admitted to the ICUs across the Northeast Georgia Health System staffed by critical care physicians. This totaled six intensive care units across 90 ICU beds. The time frame of the sample collected was between Jan 1, 2019, to June 30, 2021. Inclusion criteria for the study, in addition to requiring ICU admission, required being older than 18 years of age, having at least two procalcitonin collected, and the patient had to carry a diagnosis as labeled by ICD-10 coding for sepsis, severe sepsis, or septic shock.

Patients diagnosed with COVID-19 were excluded from the study. COVID-19 was excluded given the concern of how much variability the viral infection presented. There was also concern that the management of viral infection varied immensely during the course of the pandemic and that there was no true guideline to follow on how to manage patients infected with COVID-19. Fungal infections and other viral infections were also excluded to avoid potential confounding variables of coinfections.

The subsequent collections of procalcitonin had to be collected within 10 days from the previous draw to be considered for delta calculations. The outcomes of interest were dependent on delta procalcitonin, calculating a predictive model for mortality, morbidity, ICU length of stay, and hospital length of stay. Lastly, an observation was made if there was a change in mortality and morbidity if antibiotics were de-escalated or escalated in retrospect to the delta change in procalcitonin.

## Results

Table 1 presents the pro-cal-guided and standard treatment arms [4]. A positive delta between the initial and second pro-cal did not show statistical significance between those who expired and those who had not expired (Tables 2-3). With subsequent pro-cal collections, however, there appears to be much more of a statistical significance in relation to the disposition of the patient. A positive pro-cal collection at the second and third delta demonstrates an increased correlation to expiration. The likelihood and odds ratio also demonstrate a similar correlation, demonstrating evidence of a higher expectation of expiration of the patient with a positive delta in procalcitonin in the second and third delta (Table 4).

**Table 1:** Pro-cal-guided vs standard care for sepsis Source: Ref. [4]

	Number allocated	Time to antibiotic Cessation	Hospital Length of Stay	ICU Readmission
Pro-cal Guided	196	9	15	6
Standard care	198	11	17	12

**Table 2:** Delta procalcitonin disposition

Disposition		Delta 1 negative	Delta 1 Positive	Delta 1 Negative	Delta 2 Positive	Delta 2 Negative	Delta 3 Positive		Delta 3 Negative					
	Not Expired	Count	821	519	390	158	182		72					
		% Within Delta Positive	91.00%	78.40%	88.00%	76.70%	86.30%		77.40%					
		Standard Residual	1.7	-2	0.8	-1.2	0.4		-0.6					
	Expired	Count	81	143	53	48	29		21					
		% Within Delta Positive	9.00%	21.60%	12.00%	23.30%	13.70%		22.60%					
		Standard Residual	-4.2	4.9	-1.9	2.8	-1		1.5					

**Table 3:** Chi-square tests for delta procalcitonin disposition

Chi-Square Tests		Delta 1			Delta 2			Delta 3		
		Value	Degree of Freedom	Asymptotic significance (2-sided)	Value	Degree of Freedom	Asymptotic significance (2-sided)	Value	Degree of Freedom	Asymptotic significance (2-sided)
	Pearson Chi-Square	49.562	1	<0.001	13.33	1	<0.001	8.032	1	0.005
	Likelihood Ratio	49.063	1	<0.001	13.584	1	<0.001	8.18	1	0.004

**Table 4:** Odds ratio for delta pro-cal disposition

Odds Ratio		Delta 1			Delta 2			Delta 3		
			95% Confidence Interval			95% Confidence Interval			95% Confidence Interval	
		Value	Lower	Upper	Value	Lower	Upper	Value	Lower	Upper
	Odds Ratio for Disposition	2.793	2.082	3.747	2.235	1.451	3.444	1.83	0.98	3.418
	Not Expired	1.161	1.11	1.214	1.148	1.057	1.247	1.114	0.986	1.259
	Expired	0.416	0.323	0.536	0.513	0.36	0.731	0.609	0.367	1.009

In terms of ICU length of stays, there was a statistical significance with the expectation of a longer ICU stay if the second pro-cal had a delta positive (Table 5). The average ICU length of stay was three days longer than the other delta pro-cal deltas for both expired and not expired. Despite the increased ICU length of stay noted, there was no statistical difference in the average hospital length of stay (Table 5).

**Table 5:** ICU length of stay for delta procalcitonin

ICU Length of Stay			Delta 1			Delta 2			Delta 3		
			Mean	Standard Deviation	Standard Error Mean	Mean	Standard Deviation	Standard Error Mean	Mean	Standard Deviation	Standard Error Mean
	ICU Days	Not Expired	17.788	18.595	0.768	16.708	17.721	0.727	22.505	23.363	1.414
		Expired	16.636	15.473	0.91	19.024	17.351	1.033	20.666	15.158	1.105
	Hospital Length of Stay	Not Expired	27.37	23.642	0.991	26.68	23.476	0.981	34.28	30.478	1.879
		Expired	26	19.715	1.178	27.68	20.244	1.219	30.67	18.019	1.339

Antibiotics were examined as to whether antibiotics were changed based on the delta pro-cal (Tables 6, 7). The change was based on whether the antibiotics were broadened in coverage or narrowed. Based on the data collected, there was no statistical difference based on the change in antibiotics and the delta procalcitonin. This was seen across all the delta changes in procalcitonin, irrespective if antibiotics were changed or not.

**Table 6:** Antibiotic change for delta procalcitonin

Antibiotic Change			Delta 1		Delta 2		Delta 3	
			Not Expired	Expired	Not Expired	Expired	Not Expired	Expired
	De-Escalation	Count	57	16	96	19	41	14
		% Within Disposition	3.50%	3.00%	6.70%	4.00%	6.90%	5.80%
		Standardized Residual	0.3	-0.5	1	-1.8	0.3	-0.5
	No Escalation	Count	781	248	984	271	420	143
		% Within Disposition	47.50%	46.4%%	68.20%	56.60%	70.90%	59.60%
		Standardized Residual	0.2	-0.3	1.4	-2.4	1	-1.5
	Escalation	Count	807	271	362	189	592	240
		% Within Disposition	49.10%	50.70%	25.10%	39.50%	22.10%	34.60%
		Standardized Residual	-0.2	0.4	-2.5	4.4	-1.7	2.7

**Table 7:** Antibiotic change chi-square tests

Antibiotic Change Chi-Square Tests		Delta 1			Delta 2			Delta 3		
		Value	Degree of Freedom	Asymptotic Significance (2-sided)	Value	Degree of Freedom	Asymptotic Significance (2-sided)	Value	Degree of Freedom	Asymptotic Significance (2-sided)
	Pearson Chi-Square	0.587	2	0.746	37.66	2	<0.01	13.866	2	<0.01
	Likelihood Ratio	0.593	2	0.743	36.541	2	<0.001	13.385	2	0.001
Antibiotic Change Chi-Square Tests										

## Discussion

With this retrospective study, there is some statistical significance to suggest that, with a positive trend in delta procalcitonin, there is an increased likelihood of expiration (Tables 2-4). While there was not a statistical significance noted in overall hospital length of stay, there was an increased likelihood of longer ICU stays with a delta positive pro-cal (Table 5). There was no significance noted in antibiotic management with the patient’s disposition in relation to the change in delta pro-cal (Tables 6-7). There were, however, limitations in this statistical entity given that antibiotics will vary as what is viewed as a de-escalation versus escalation may vary from clinician to clinician. The evaluation of broadening and narrowing of antibiotics and how it could affect delta pro-cal could be better delineated with a stricter antibiotic standardization with antibiotics placed in tiers to determine if an escalation or de-escalation took place and their relationship to delta procalcitonin.

With the important randomized control trials listed earlier, there have been several studies in between these breakthrough trials through retrospective studies to observe the usefulness of the use of procalcitonin in the management of septic patients [8-12]. This has led to the possibility of a gold standard biomarker to assist the critical care teams in the management of patients who may be suspected of having sepsis and those having sepsis [1-4,8-15]. These studies specifically observed the patients at the critical care level of medicine given the mortality and morbidity rate of severe sepsis within the intensive care unit [8-12]. The consensus among these studies demonstrates the usefulness of pro-cal data to guide the use of antibiotics in patients with concern for bacterial infections or even those patients with sepsis [8-12].

Given the increased likelihood of mortality and morbidity with an up trending of procalcitonin, it would be prudent to be aggressive with source control and managing of antimicrobials in consortium with infectious disease experts. This would include escalation of antimicrobials as deemed appropriate [13-15]. We recognize that our study did not show an escalation of antibiotics with the rise in pro-cal, leading to a statistically significant improvement in mortality. This may simply mean that the absence of a meaningful decrease in pro-cal may portend higher mortality despite change in antibiotics, as alluded to in the MOSES study but also allow for an opportunity to further study how antibiotics should be managed based on delta pro-cal [15]. Overall, as alluded to by earlier studies conducted using procalcitonin to guide sepsis management, conducting serial pro-cal may be beneficial to patient care in the case of sepsis [1,4,15]. Not only would this improve morbidity and mortality, but also there is a clear financial benefit as well to hospital systems based on the length of ICU stay, and the overall hospital stay may be shortened.

## Conclusions

While ESR and CRP have been used for acute processes as biomarkers, procalcitonin should be viewed as the biomarker for sepsis because of its sensitivity and specificity toward bacterial infections as previously mentioned. This study puts into the spotlight how serial procalcitonins can be used to guide therapy within critically ill patients. Critically ill patients have a high risk of clinical deterioration and using a marker to detect a potential clinical deterioration can improve mortality and morbidity. The studies previously done primarily looked at the use of pro-cal as a means of guiding antibiotics primarily as a means of not using or discontinuing antibiotic use. None have looked at its use for the escalation of antibiotics or predicting mortality and morbidity.

This study's purpose was to determine an association of up-trending procalcitonin with any escalation of antibiotics with mortality. We found that there is a signal of uptrending procalcitonin correlating with an increase in mortality but neither correlates with the escalation of antibiotics. Further studying of procalcitonin, specifically the delta differences between collections, may lead to the identification of a gold standard biomarker for the management of sepsis in patients.
